# Correlations among Brain Gray Matter Volumes, Age, Gender, and Hemisphere in Healthy Individuals

**DOI:** 10.1371/journal.pone.0022734

**Published:** 2011-07-27

**Authors:** Yasuyuki Taki, Benjamin Thyreau, Shigeo Kinomura, Kazunori Sato, Ryoi Goto, Ryuta Kawashima, Hiroshi Fukuda

**Affiliations:** 1 Division of Developmental Cognitive Neuroscience, Institute of Development, Aging and Cancer, Tohoku University, Sendai, Japan; 2 Department of Nuclear Medicine and Radiology, Institute of Development, Aging and Cancer, Tohoku University, Sendai, Japan; 3 Department of Functional Brain Imaging, Institute of Development, Aging and Cancer, Tohoku University, Sendai, Japan; 4 Smart Ageing International Research Centre, Institute of Development, Aging and Cancer, Tohoku University, Sendai, Japan; Beijing Normal University, Beijing, China

## Abstract

To determine the relationship between age and gray matter structure and how interactions between gender and hemisphere impact this relationship, we examined correlations between global or regional gray matter volume and age, including interactions of gender and hemisphere, using a general linear model with voxel-based and region-of-interest analyses. Brain magnetic resonance images were collected from 1460 healthy individuals aged 20–69 years; the images were linearly normalized and segmented and restored to native space for analysis of global gray matter volume. Linearly normalized images were then non-linearly normalized and smoothed for analysis of regional gray matter volume. Analysis of global gray matter volume revealed a significant negative correlation between gray matter ratio (gray matter volume divided by intracranial volume) and age in both genders, and a significant interaction effect of age × gender on the gray matter ratio. In analyzing regional gray matter volume, the gray matter volume of all regions showed significant main effects of age, and most regions, with the exception of several including the inferior parietal lobule, showed a significant age × gender interaction. Additionally, the inferior temporal gyrus showed a significant age × gender × hemisphere interaction. No regional volumes showed significant age × hemisphere interactions. Our study may contribute to clarifying the mechanism(s) of normal brain aging in each brain region.

## Introduction

Previous studies using magnetic resonance (MR) imaging have demonstrated a negative correlation between global or regional gray matter volume and age by applying cross sectional [1]–[16] and longitudinal designs [10], [17]–[23]. Evaluating the correlation between gray matter volume and age is important when distinguishing neurodegenerative diseases from normal aging, particularly for neurodegenerative diseases such as Alzheimer's disease (AD), which show regional gray matter volume decline compared with age-matched healthy subjects [24], [25]. Regional gray matter volume in several regions is associated with cognitive functions, such as attention and executive function [26]–[30]. Thus, examining the correlation between brain gray matter volume and age might help reveal mechanism(s) of normal brain aging and might be useful in distinguishing normal from pathological aging.

To examine any correlation between regional gray matter volume and age, interactions of gender and hemisphere should be considered. Research on the effect of the interaction of gender and age on regional gray matter volume has shown significant age × gender interaction effects on the cortical thickness of the bilateral dorsal frontal region and the right temporal regions, whereas no such interaction was significant for the bilateral temporoparietal regions [31]. Additionally, significant age × gender interactions were also found for the hippocampus and the fusiform gyrus [32]. These studies showed that the magnitude of gender differences varied with age. However, although several studies have focused on the effect of age × gender interactions on regional gray matter volume, the findings of these studies were restricted to specific areas, such as the cortical areas [31], thalamus [11], 13 manually traced cerebral regions of interest [32], or to limited age groups, such as elderly subjects [16]. Thus, prior research has not clarified the effect of age × gender interactions on the global and regional gray matter volumes of each gyral structure in the cerebrum, on deep gray matter structures, such as the thalamus and caudate nucleus, or on each lobe of the cerebellum. One longitudinal study on age × hemisphere interactions in healthy subjects showed that gray matter volume decline was more substantial in the right hemisphere of healthy elderly people [10], whereas other studies have suggested that hemispheric atrophy was more severe in the left hemisphere, especially in men [33], [34]. Research has also shown that such gray matter regions as the entorhinal cortex and temporoparietal cortices of the left hemisphere were affected earlier and more severely than were those in the right hemisphere in patients with Alzheimer's disease [35]. However, no reported study has revealed age × hemisphere or age × gender × hemisphere interaction effects on the regional gray matter volume of each gyral structure of the cerebrum, on such deep gray matter structures as the thalamus and caudate nucleus, and on each lobe of the cerebellum.

Cross-sectional and longitudinal designs are useful for analyzing the correlation between age and gray matter volume. Recent studies have primarily reported a correlation between age and gray matter volume using longitudinal analyses [10], [17]–[23], because a longitudinal design can overcome confounding factors, such as nutrition and medical care, from which cross-sectional studies suffer. Additionally, because there are inter-individual differences in gray matter volume, the actual gray matter volume change with age is underestimated when using a cross-sectional design versus a longitudinal design [19]. However, a longitudinal study includes methodological limitations too, such as selection bias of the subjects, in which subjects who are not healthy or who are not socio-economically sufficient may be dropped in the follow-up study. Furthermore, it is more difficult to collect data from a large number of subjects compared with a cross-sectional design and it is also difficult to maintain the MR scanner in exactly the same state over several years. Thus, not only longitudinal designs, but also cross-sectional designs, are necessary to reveal correlations between gray matter volume and age.

Using a cross-sectional design, the purpose of this study was to analyze the main effect of age and the effect of age × gender interactions on global and regional gray matter volumes as well as to examine the effect of age × gender × hemisphere interactions on regional gray matter volume in a large number of healthy individuals within a wide age range. First, we analyzed the correlation between global gray matter volume and age using MR images of 1460 healthy Japanese subjects, aged 20–69 years, using a fully automated image processing technique. We also analyzed the correlation between global white matter volume, cerebrospinal fluid (CSF) space volume, and age because such results might aid in understanding the biological meaning of changes in gray matter volume. We then used a general linear model to assess the main effects of age and age × gender interactions on global gray matter volume. Second, we analyzed the correlation between age and regional gray matter volume using parcellation analysis, in which the gray matter region of the cerebrum and the cerebellum was divided into 32 parcels corresponding to anatomical structures in each hemisphere. To accomplish this, we analyzed the main effects of age, gender, and hemisphere as well as age × gender, age × hemisphere, and age × gender × hemisphere interactions on regional gray matter volume in each parcel, using a general linear model.

In this study, we focused on the main effect of age and the effect of age × gender and age × gender × hemisphere interactions on gray matter volume in an analysis of regional gray matter volume. We hypothesized that regional gray matter volumes of the association cortex of the frontal and parietal lobes, such as the middle frontal gyrus and the inferior parietal lobe, would show larger declines with age than the gray matter volumes of the limbic and paralimbic system. This hypothesis was derived from the results of recent studies, which have shown that later-maturing cortical regions, such as prefrontal cortex, and superior and inferior parietal lobules, are more vulnerable to age-related morphological changes than the limbic and paralimbic system [6], [9]. We also hypothesized that there would be significant age × gender interactions for global and regional gray matter volumes, because several factors that affect gray matter volume, such as sex steroid hormones [36], [37] and cerebrovascular risk factors, such as obesity and heavy alcohol drinking [12], [38], [39], vary with age in different ways in men and women. We did not have a clear hypothesis for the age × hemisphere or age × gender × hemisphere interactions.

## Methods

### Subjects

Written informed consent was obtained from each subject prior to MRI and after a full explanation of the purpose and procedures of the study, which were consistent with the Declaration of Helsinki (1991). Additionally, written informed consent was obtained from each subject and his/her parents for subjects under 20 years of age prior to MRI and after a full explanation of the purpose and procedures of the study. Approval for the experiments was obtained from the institutional review board of Tohoku University.

All subjects were Japanese people recruited through the Aoba Brain Imaging Project in Sendai, Japan. The Aoba Brain Imaging Project was conducted to create a database of normal Japanese brain images [40]. The purpose of the project was announced through the mass media, which was used to recruit volunteers, who called our project center to state their interest in participating. During a preliminary telephone interview, we excluded volunteers with a past or present history of any malignant tumor, head trauma, cerebrovascular disease, epilepsy, or any psychiatric disorder. As a result, we collected brain MR images of 1,637 subjects (age range 12–81 years in men, 12–80 in women). All MR images were inspected by radiologists, and images revealing any of the following were excluded: brain tumors of any kind, major infarctions (except lacunar infarction), and hemorrhages (observed in low-intensity areas in T2-weighted images). Correlations between global/regional gray matter volumes and age as well as the main and interaction effects involving age, gender, and hemisphere determined using a general linear model might be affected by age (especially at both endpoints of the continuum). However, relatively few subjects were aged under 20 or over 70. Thus, we included only those subjects aged 20–69 in this study. As a result, we analyzed brain images of 1460 subjects, including 702 men and 758 women aged 20–69 years. Characteristics of the subjects are shown in Table 1. Additionally, Student's *t*-test was performed on each factor by gender, as shown in Table 1. Past or present smoking, drinking habits, hypertension, diabetes mellitus, hypercholesterolemia, and ischemic heart disease were scored as ‘1’ for an affirmative and ‘0’ for a negative response to each question. Subjects who consumed alcohol more than once a week were classified as having a drinking habit. We performed Mann–Whitney *U*-tests to analyze gender differences for these factors. We divided subjects into age groups according to decade (third decade (age range: 20–29), fourth decade (age range: 30–39), fifth decade (age range: 40–49), sixth decade (age range: 50–59), and seventh decade (age range: 60–69)) and analyzed the correlation between age group and gender and hemisphere to identify significant interactions.

**Table 1:** Subject characteristics.

	Men (*N* = 702)	Women (*N* = 758)	*P*
Age			
Whole group (20–69 years)	45.11±14.64	46.08±13.29	0.204
Third decade age group (20–29 years)	23.4±2.8 (*n* = 161)	24.8±2.9 (*n* = 130)	<0.001
Fourth decade age group (30–39 years)	34.6±2.8 (*n* = 113)	34.5±2.7 (*n* = 117)	0.807
Fifth decade age group (40–49 years)	44.9±3.0 (*n* = 134)	45.7±2.7 (*n* = 163)	0.014
Sixth decade age group (50–59 years)	54.6±2.9 (*n* = 162)	53.8±2.9 (*n* = 220)	0.013
Seventh decade age group (60–69 years)	64.1±2.9 (*n* = 132)	63.8±2.6 (*n* = 128)	0.343
Body mass index	23.51±3.24	22.26±2.97	<0.001
Systolic blood pressure (mmHg)	129.1±15.9	124.6±17.6	<0.001
Diastolic blood pressure (mmHg)	79.0±11.2	75.0±12.0	<0.001
Past or present smoking habit (%)	61.1	17.0	<0.001
Past or present drinking habit (%)	59.4	18.1	<0.001
Hypertension (%)	13.2	9.3	0.027
Diabetes mellitus (%)	3.8	1.4	0.005
Hypercholesterolemia (%)	6.3	9.8	0.022
Ischemic heart disease (%)	1.1	0.3	0.062

Body mass index is calculated by weight (kg)/height (m^2^). Values for age, body mass index, and systolic and diastolic blood pressures are expressed as mean ± SD.

### Image acquisition

Brain MR images of each subject were taken using the same 0.5 T MR scanner (Signa contour, GE-Yokogawa Medical Systems, Tokyo) with two different pulse sequences: (1) 124 contiguous, 1.5-mm-thick axial planes of three-dimensional T1-weighted images (spoiled gradient recalled acquisition in steady state: repetition time (TR), 40 ms; echo time (TE), 7 ms; flip angle, 30°; voxel size, 1.02×1.02×1.5 mm); (2) 63 contiguous, 3-mm-thick axial planes of gapless (using interleave) proton probability density (PD) weighted images/T2-weighted images (dual echo fast spin echo: TR, 2860 ms; TE, 15/120 ms; voxel size, 1.02×1.02×3 mm). The T1-weighted images were used for image analysis, and the PD-weighted images/T2-weighted images were used for clinical evaluation.

### Image analysis of global gray matter, white matter, and CSF space volume

After the image acquisition, all T1-weighted MR images were analyzed using Statistical Parametric Mapping 2 (SPM2; Wellcome Department of Cognitive Neurology, London, UK) [41] in Matlab (MathWorks, Natick, MA) and part of the Matlab program “cg_vbm_optimized” (http://dbm.neuro.uni-jena.de/vbm.html). First, the T1-weighted MR images were transformed into Talairach stereotaxic space [42] using a 12-parameter affine transformation [43] and the same template image, namely the ICBM 152 template derived from the Montreal Neurological Institute, and derived from 152 normal subjects and which approximates Talairach space [42]. Then, tissue segmentation from the transformed images to the gray matter, white matter, CSF space, and non-brain was performed using the SPM2 default segmentation procedure. Next, the segmented gray matter images were non-linearly normalized to the gray matter template of SPM2 using 7×8×7 non-linear basis functions in three orthogonal directions. These normalization parameters were reapplied to the T1-weighted whole brain structural images of each subject to perform spatial normalization. The normalized T1-weighted images were segmented into gray matter, white matter, and CSF space. The normalized, segmented gray matter images were then modulated by calculating the Jacobian determinants, derived from the special normalization step, and multiplying each voxel by the relative change in volume, as in the method of Good *et al.* [5]. This modulation step was performed to correct for volume change in the non-linear normalization. The volumes of global gray matter, white matter, and CSF space were calculated using segmented and modulated images by adding a value derived from the voxel volume and multiplied by the value of each voxel. We also calculated the global gray matter volume of the cerebrum and the cerebellum in each hemisphere by applying a region of interest (ROI) for each hemisphere, created using the “WFU_PickAtlas” (http://www.fmri.wfubmc.edu/download.htm) [44], [45]. We calculated the ratio of gray matter volume in each hemisphere, adjusted for the intracranial volume of each subject to adjust for differences in head size, and defined this measure as the gray matter ratio. Intracranial volume was calculated by summing gray matter volume, white matter volume, and CSF space volume. We defined the white matter ratio and CSF space ratio in the same manner.

### Image analysis of regional gray matter volumes

**Figure 1:**
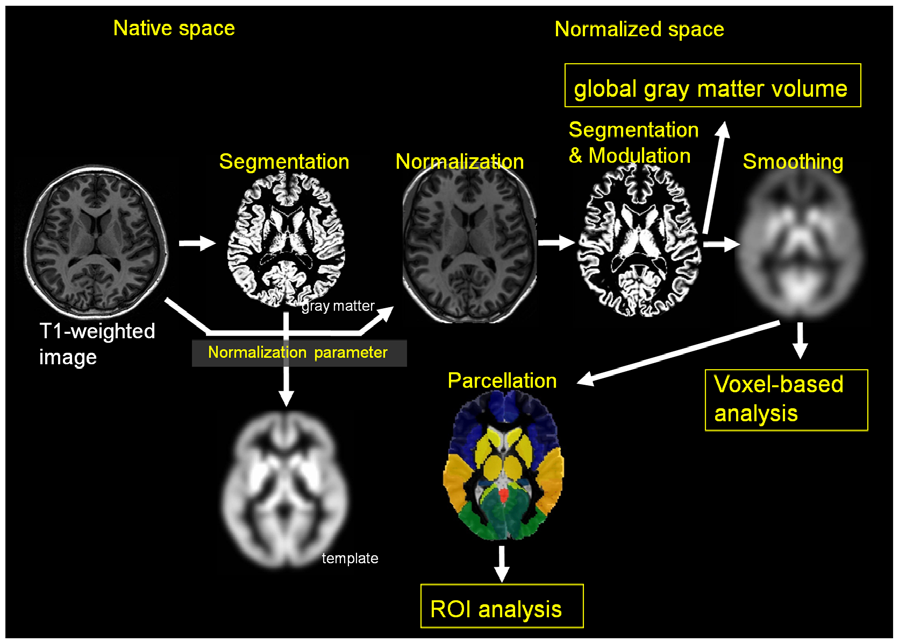
Schema of brain MR image analysis for global and regional gray matter volume.

To calculate regional gray matter volumes (the volumes of each parcel), the normalized, segmented, and modulated gray matter images, derived from the image analysis of global gray matter volume, were then smoothed by convoluting a 12-mm-FWHM isotropic Gaussian kernel. This smoothing step was used to remove individual variations in gyral anatomy and to render data more normally distributed by the central limit theorem. We set the ROI to cover the entire gray matter structural region of the cerebrum, the deep gray matter structures, or each lobe of the cerebellum in both hemispheres using the “WFU_PickAtlas” [44], [45] to obtain the gray matter volume of each region bilaterally. The location of each ROI is shown in Table S1. The mean and standard deviation of each regional gray matter volume and the percentage of annual decrease estimated by the slope of the linear regression between regional gray matter volume and age are also shown in Table S1. All regional gray matter volumes, except for that of the bilateral posterior cingulate cortex (PCC) in men, correlated significantly with age (P<0.001 in all correlations). While initial analysis of the gray matter volume of the PCC in men showed a substantial correlation with age (left, P = 0.006; right, P = 0.045), the significance of this correlation did not survive after correcting for multiple comparisons (*P*<0.05/32 = 0.002). A schematic of the image analysis of global and regional gray matter volumes is shown in Figure 1.

### Statistical analysis of global gray matter, white matter, and CSF space volume

Statistical analyses of global gray matter, white matter, and CSF space volume were conducted separately. Correlations between gray matter ratio, white matter ratio, and CSF space ratio and age were estimated using first-, second-, and third-order polynomial functions, and we determined the best-fit model by selecting the function that showed the smallest Akaike information criterion (AIC) [46]. Next, we analyzed the effects of age, gender, and hemisphere, and the interaction effects of age × gender, age × hemisphere, and age × gender × hemisphere on gray matter ratio using a general linear model. These effects and interactions were estimated using effect size by calculating the partial *η* ^2^. Next, to examine the age × gender interaction, we divided the subjects into age groups by decade (third, fourth, fifth, sixth, and seventh decade), and performed unpaired t-tests between data from males and females within each age group. If the mean age differed between males and females of the same age group, we applied ANCOVAs instead of unpaired t-tests to adjust for these age differences. The significance level was set at *P*<0.05.

### Statistical analysis of regional gray matter volume

**Figure 2:**
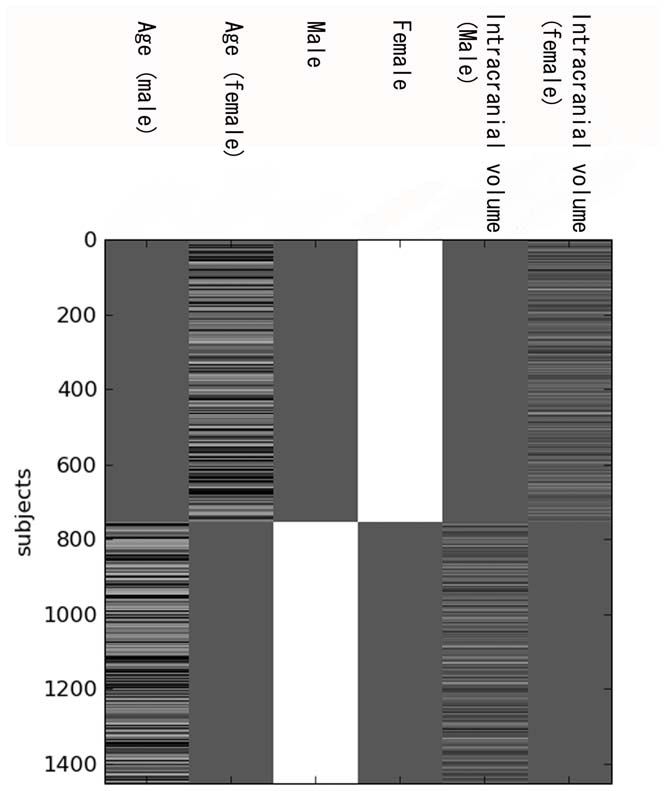
Design matrix of regional gray matter volume using statistical parametric mapping.

To analyze the effects of age, gender, and hemisphere and the interaction effects of age × gender, age × hemisphere, and age × gender × hemisphere on regional gray matter volume, we performed voxel-based regressions using the statistical parametric map (SPM) framework. In that framework, a single multi-linear model of the age across subjects was designed and then estimated at every voxel of realigned brains using a least square regression (i.e., assuming Gaussian-distributed errors). The statistical significance of the fit at every location was assessed to yield brain maps of the effects. We created a design matrix, which included two pairs of regressors (i.e., age and intercept), one for each gender, in order to linearly regress the age onto male and female groups independently. Additionally, the total intracranial brain volume was added as a covariate of non-interest for each group. Age and intracranial volume regressors were centered on their group mean. The resulting design matrix is illustrated in Figure 2. Using this design allowed us to test for a linear relationship of age to gray matter volume all over the brain, as well as a difference in slope for the male and female groups. Specifically, the former was tested by assessing the average slope parameter of the two fitted lines using the {(age (male) + age (female))/2} contrast, whereas the latter assessed the difference of those slope parameters using the {(age (male) – age (female))/2} contrast. Furthermore, the model allowed us to investigate the potential interaction of age and gender with gray matter asymmetries by fitting it over half-brain images made from the difference between right and left voxels of the gray matter volume images in MNI space, following the idea of [47]. Those asymmetry images were computed by simple voxel-wise subtraction of the right hemisphere and the left hemisphere mirrored over the sagittal plane. The resulting images are illustrated in the right part of Figure 2. The linear hypotheses were assessed using the same contrasts as before. That is, the {(age (male) + age (female))/2} contrast was used to test for a linear relationship between age and asymmetry within each gender group, whereas the {(age (male)−age (female))/2} contrast assessed the difference between those two age-to-asymmetry slopes (i.e., age × gender × hemisphere interaction). The significance level was set at *P*<0.05, and voxel-based analyses were corrected for multiple comparisons by the family-wise error rate.

Next, we analyzed the effects of age, gender, and hemisphere, and the interaction effects of age × gender, age × hemisphere, and age × gender × hemisphere on regional gray matter volume in each structure. These effects were estimated using a general linear model in each ROI. We calculated main effects of age, gender, hemisphere, and interaction effects of age × gender, age × hemisphere, and age × gender × hemisphere on regional gray matter volume in each ROI. We also used intracranial volume as a covariate to exclude the effect of the intracranial volume. These effects and interactions were estimated by calculating the partial *η* ^2^. Intracranial volume was used as a covariate to adjust for individual differences in this variable. Separate correlations between age and regional gray matter volume were performed in each ROI. We determined the significance level using the Bonferroni correction. The total number of ROIs was 32 in each hemisphere; thus, the significance level was set at *P*<0.05/32 = 0.002 in all statistical analyses of regional gray matter volume in ROI analysis. Finally, correlations between regional gray matter volume in each structure and age were estimated using first-, second-, and third-order polynomial functions, and we determined the best-fit model by selecting the function that showed the smallest Akaike information criterion (AIC) [46] in each ROI separately. These results are shown in Table S2.

## Results

### 1. Global gray matter volume, white matter volume, and CSF space volume

#### 1.1. Correlation of ratio of each segment with age

**Figure 3:**
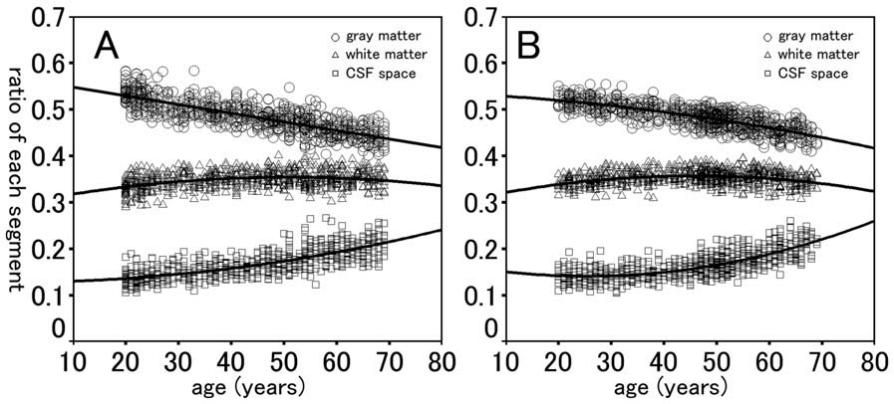
Relationship between ratios of gray matter, white matter, and CSF fluid space segments and age for men (A), and women (B). Circles represent gray matter ratio, triangles represent white matter ratio, and squares represent CSF space ratio.

Applying AIC, we determined that the correlation between age and gray matter ratio was significant and best fit by a first-order polynomial function in men (*R* ^2^ = 0.643; *p*<0.001) and a second-order polynomial function in women (*R* ^2^ = 0.624; *p*<0.001). The correlation between age and white matter ratio was also significant and best fit with a second-order polynomial function in both men (*R* ^2^ = 0.121; *p*<0.001) and women (*R* ^2^ = 0.101; *p*<0.001). The correlation between age and CSF space ratio was significant and best fit with a second-order polynomial function in both men (*R* ^2^ = 0.583; *p*<0.001) and women (*R* ^2^ = 0.561; *p*<0.001). The correlations between age and gray matter ratio, white matter ratio, and CSF space ratio are shown in Figures 3A for men and 3B for women.

#### 1.2. Main effects of age, and interaction effects of age × gender, age × hemisphere, and age × gender × hemisphere on gray matter ratio, white matter ratio, and CSF space ratio

**Figure 4:**
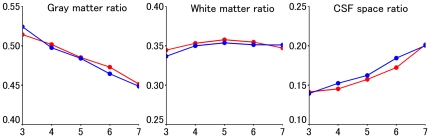
Relationship between gray matter ratio, white matter ratio, and CSF space ratio and age group, moving from left to right. Blue lines and markers indicate men, and red lines and markers indicate women. In each graph, the vertical axis indicates regional gray matter volume (no units), and the horizontal axis refers to subjects in the third (aged 20–29), fourth (aged 30–39), fifth (aged 40–49), sixth (aged 50–59), and seventh (aged 60–69) decades of life, respectively, moving from left to right.

A significant main effect of age on gray matter ratio was found (partial *η* ^2^, 0.619; *P*<0.001). Additionally, we found a significant interaction effect of age × gender on gray matter ratio (partial *η* ^2^, 0.054; *P*<0.001). There was no significant interaction effect of age × hemisphere (partial *η* ^2^, <0.001; *P* = 1.0) or age × gender × hemisphere (partial *η* ^2^, <0.001; *P* = 1.0) on gray matter ratio. Regarding the white matter ratio, there was a significant main effect of age (partial *η* ^2^, 0.102; *P*<0.001) and a significant interaction effect of age × gender (partial *η* ^2^, 0.046; *P*<0.001). However, there was no significant interaction effect of age × hemisphere (partial *η* ^2^, <0.001; *P* = 1.0) or age × gender×hemisphere (partial *η* ^2^, <0.001; *P* = 1.0) on white matter ratio. Regarding CSF space, there was a significant main effect of age (partial *η* ^2^, 0.535; *P*<0.001) and a significant interaction effect of age × gender (partial *η* ^2^, 0.040; *P*<0.001) but there was no significant interaction effect of age × hemisphere (partial *η* ^2^, <0.001; *P* = 1.0) or of age × gender × hemisphere (partial *η* ^2^, <0.001; *P* = 1.0). Because there were significant interaction effects of age × gender on gray matter ratio, white matter ratio, and CSF space ratio, we examined the correlation between age group and gray matter ratio, white matter ratio, and CSF space ratio in men and women (Fig. 4). After adjusting for differences in mean age, the gray matter ratio was significantly higher in men than in women in their third decade of life (*t* = 2.791; *P* = 0.006). However, no significant gender difference was found in this measure for subjects in their fourth (*t* = 1.662; *P* = 0.098) or fifth (*t* = 0.501; *P* = 0.617) decades. The gray matter ratio was significantly higher in women than in men, adjusting for age (*t* = 3.373; *P*<0.001) for subjects in their sixth decade, while no significant gender differences were found in this measure for subjects in their seventh decade (*t* = 1.239; *P* = 0.217).

### 2. Regional gray matter volume

#### 2.1. Main effects of age, and interaction effects of age × gender, age × hemisphere, and age × gender × hemisphere on regional gray matter volumes

**Figure 5:**
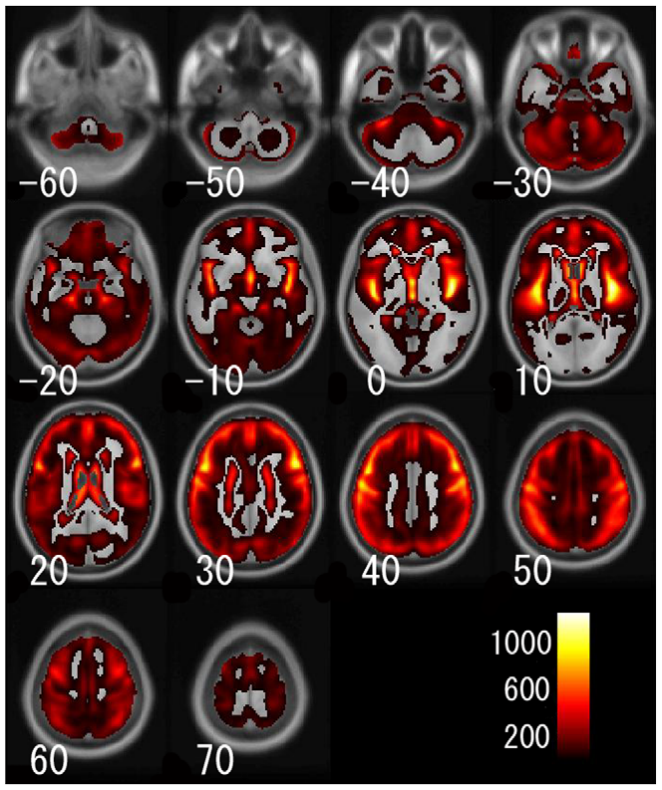
Gray matter regions that showed a significant main effect of age on regional gray matter volume. The left side of the image represents the left side of the brain. Color scales indicate the *F*-score. The number at the bottom of the left side of each image indicates the value of the *z*-axis in the Talairach stereotaxic space.

**Figure 6:**
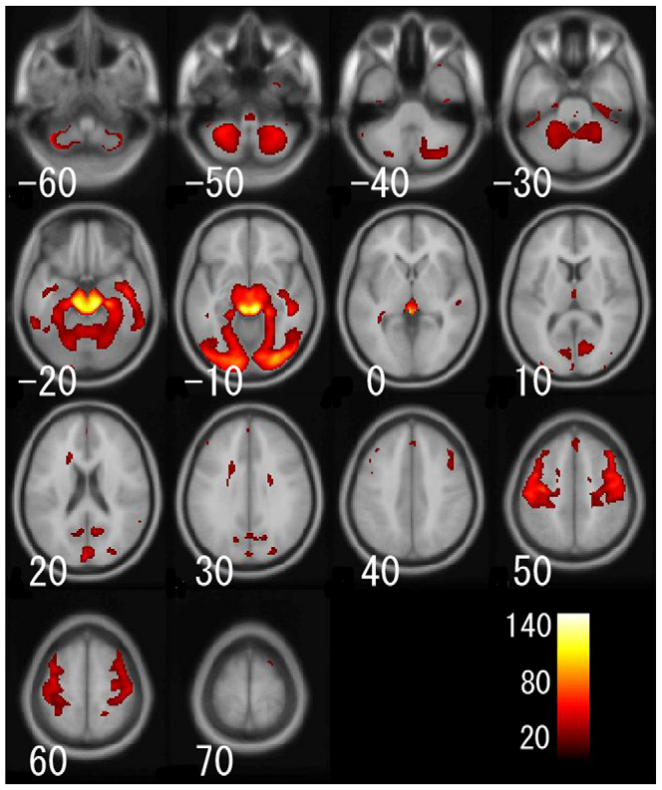
Gray matter regions that showed a significant age × gender interaction on regional gray matter volume. Details are the same as in Figure 4.

**Figure 7:**
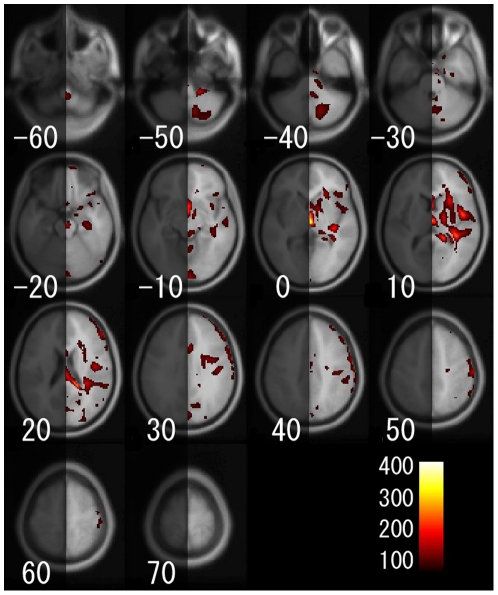
Gray matter regions that showed a significant age × hemisphere interaction on regional gray matter volume. Details are the same as in Figure 4.

**Figure 8:**
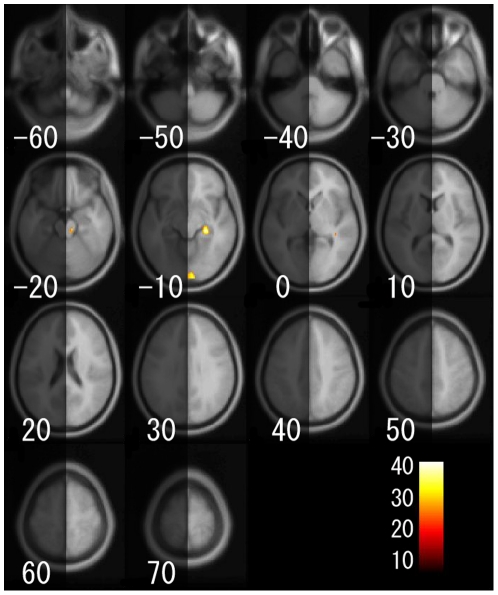
Gray matter regions that showed a significant age × gender × hemisphere interaction on regional gray matter volume. Details are the same as in Figure 4.

For the voxel-based analysis, Figure 5 shows a main effect of age on regional gray matter volume. Figures 6, 7, and 8 show the interaction effects of age × gender, age × hemisphere, and age × gender × hemisphere on regional gray matter volumes, respectively. Additionally, the gray matter regions and coordinates of Talairach space of local maxima showing significant main effects of age, interaction of age × gender, age × hemisphere, and age × gender × hemisphere are shown in Table S3, S4, S5, and S6. For the ROI analysis, Table S7 shows the magnitude of the main effects of age, and of the interaction effects of age × gender, age × hemisphere, and age × gender × hemisphere on regional gray matter volumes for each brain region.

Regarding the main effect of age on regional gray matter volume, as shown in Figure 5 and Table S7, almost all gray matter regions, especially the pre/postcentral gyri, the frontal lobes, including the superior, middle, and inferior frontal gyri, the insula, and the inferior parietal lobule, showed larger main effects for age than did other regions. On the other hand, several regions, such as the posterior aspect of the cingulate gyrus, the thalamus, the inferior temporal gyrus, the posterior lobes of the cerebellum, and the parahippocampal gyrus, showed significant, but smaller, main effects for age than did other regions.

Several regions showed significant age × gender interactions on regional gray matter volume, as shown in Figure 6 and Table S7. In particular, gray matter regions, including the fusiform gyrus, inferior occipital gyrus, cuneus, parahippocampal gyrus, and lingual gyrus, showed high effect sizes for age × gender interactions in the ROI analysis. In the voxel-based analysis, not only the fusiform gyrus and the inferior occipital gyrus but also the bilateral dorsolateral prefrontal cortex showed significant age × gender interaction.

As for the age × hemisphere interaction on regional gray matter volume, as shown in Figure 7 and Table S7, although no region showed a significant age × hemisphere interaction in the ROI analysis, voxel-based analysis showed a significant age × hemisphere interaction in several regions such as the insula, prefrontal cortex, and the posterior lobe of the cerebellum.

As shown in Figure 8 and Table S7, the inferior temporal gyrus showed a significant age × gender × hemisphere interaction on regional gray matter volume in ROI analysis, and the hippocampus and the inferior occipital gyrus showed significant age × gender × hemisphere interaction in voxel-based analysis.

#### 2.2. Correlation between regional gray matter volume and age in men and women for each brain region

**Figure 9:**
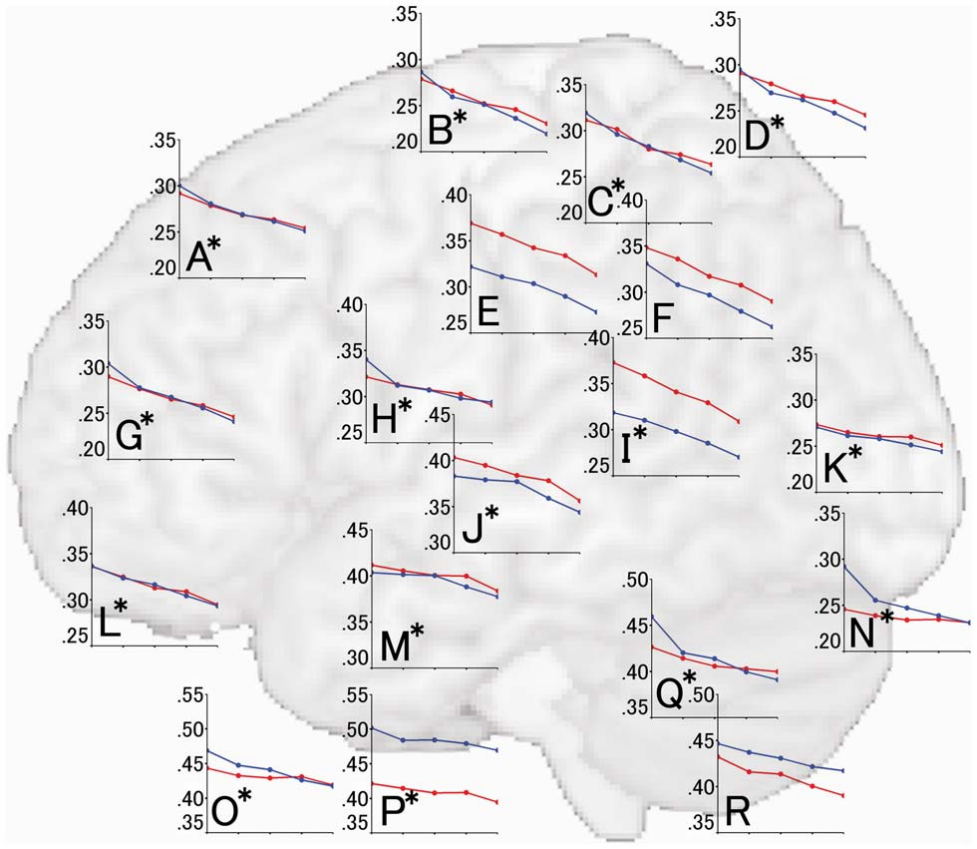
Correlations between age group and regional gray matter volumes in the superior frontal gyrus (A), precentral gyrus (B), superior parietal lobule (C), postcentral gyrus (D), supramarginal gyrus (E), inferior parietal lobule (F), middle frontal gyrus (G), paracentral lobule (H), angular gyrus (I), superior temporal gyrus (J), middle occipital gyrus (K), inferior frontal gyrus (L), middle temporal gyrus (M), inferior occipital gyrus (N), left inferior temporal gyrus (O), right inferior temporal gyrus (P), anterior lobe of the cerebellum (Q), and posterior lobe of the cerebellum (R). Graphs of both the left and right hemispheres of the inferior temporal gyrus are shown because of the age × gender × hemisphere interaction in the region. In each graph, the vertical axis indicates regional gray matter volume (no units), and the horizontal axis refers to subjects in the third (aged 20–29), fourth (aged 30–39), fifth (aged 40–49), sixth (aged 50–59), and seventh (aged 60–69) decades of life, respectively, moving from left to right. Blue and red lines indicate data obtained from men and women, respectively. To facilitate comparisons of the rate of gray matter volume decline in each region, the range of the vertical axis was set at 0.15, with the exception of the left and right inferior temporal gyri (in which the range was set at 0.20). Each graph is superimposed onto a brain MR image of the lateral view that nearly corresponds to the anatomical location. Asterisk in each graph indicates a region that shows significant age by gender interactions.

**Figure 10:**
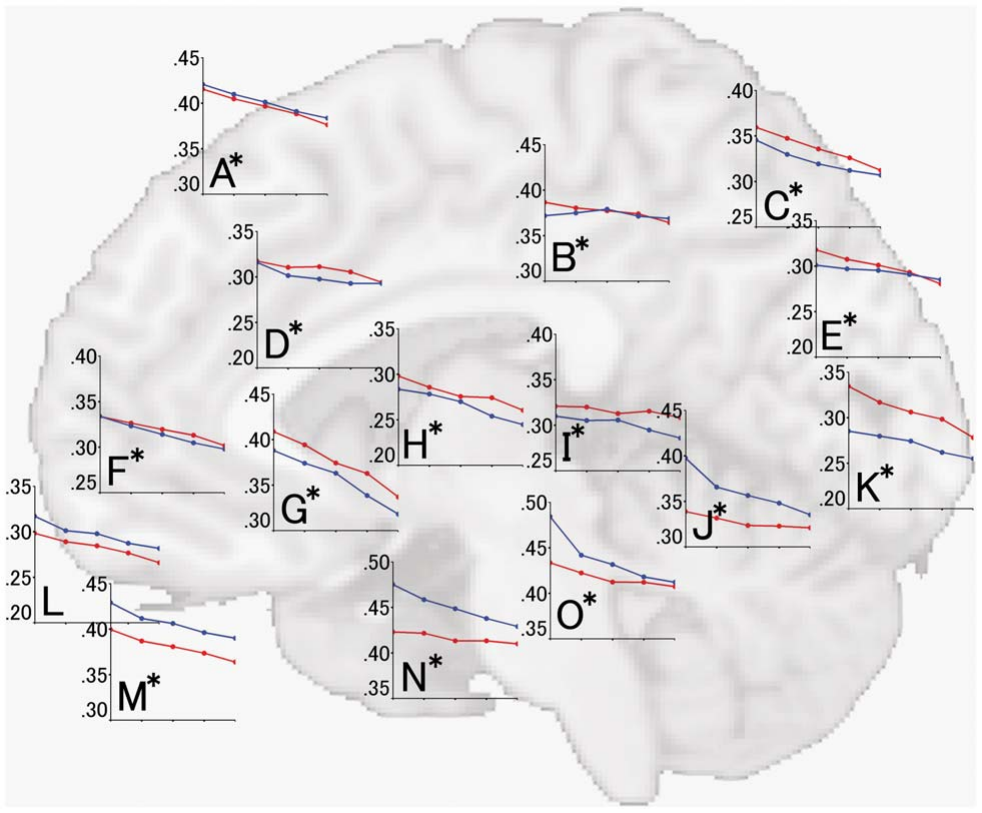
Correlations between age group and regional gray matter volumes for the medial aspect of the superior frontal gyrus (A), posterior cingulate cortex (B), precuneus (C), cingulate cortex (D), cuneus (E), anterior cingulate cortex (F), insula (G), caudate nucleus (H), thalamus (I), lingual gyrus (J), superior occipital gyrus (K), orbital gyrus (L), rectal gyrus (M), parahippocampal gyrus (N), and fusiform gyrus (O). Details are the same as in Figure 4. Each graph in each parcel is superimposed onto a brain MR image of the medial view that nearly corresponds to the anatomical location.

Because most regions showed significant age × gender interactions and the inferior frontal gyrus showed a significant age × gender × hemisphere interaction, we examined these interactions by dividing the subjects into decade-long age groups and then analyzed the correlation between age group and regional gray matter volume for each brain region in men and women. Correlations between regional gray matter volume and age group in men and women for each region are shown in Figures 9 and 10. With the exception of the supramerginal gyrus (Graph E in Fig. 9), the inferior parietal lobule (Graph F in Fig. 9), the orbital gyrus (Graph L in Fig. 10), and the posterior lobe of the cerebellum (Graph R in Fig. 9), all regions showed significant age × gender interactions. Several regions that showed effects for age × gender interactions, such as the fusiform gyrus (Graph O in Fig. 10), the inferior occipital gyrus (Graph N in Fig. 9), the parahippocampal gyrus (Graph N in Fig. 5), and the lingual gyrus (Graph J in Fig. 10), showed steep decreases in gray matter volumes in men, especially in younger men, whereas no such age-related decrease was found in women. However, a greater age × gender interaction effect was found for gray matter volume in the cuneus, where women demonstrated a steeper age-related decrease in volume than did men (Graph E in Fig. 10). The gray matter volume of the inferior temporal gyrus showed a significant age × gender × hemisphere interaction effect. When the effect of an age × gender interaction was further considered in each hemisphere, the left inferior temporal gyrus showed a significant age × gender interaction effect on gray matter volume (Graph O in Fig. 9; partial *η* ^2^, 0.063; *P*<0.001), that was not present in the right inferior temporal gyrus (Graph P in Fig. 9; partial *η* ^2^, 0.044; *P* = 0.103).

## Discussion

To our knowledge, we have produced three novel results using a large dataset of healthy individuals over a wide age range. First, application of the AIC identified a correlation between age and gray matter ratio as significant, with the correlation best fit by a first-order polynomial function in men and a second-order polynomial function in women. As hypothesized, we found a significant interaction effect of age × gender on the gray matter ratio. Second, as hypothesized, the regional volumes of gray matter in brain areas within the association cortex of the frontal and parietal lobes, such as the middle frontal gyrus and the inferior parietal lobe, showed steeper declines with aging, compared with the volumes of gray matter in regions of the limbic and paralimbic system. Third, we found significant age × gender interactions in most gray matter regions, as hypothesized. On the other hand, we found no significant age × hemisphere interactions for any region, but did find a significant age × gender × hemisphere interaction effect on the gray matter volume of the inferior temporal gyrus.

### 1. Global gray matter volume

The AIC analysis showed that the correlation between age and gray matter ratio was significant and was best fit by a first-order polynomial function in men and a second-order polynomial function in women. We also found a significant interaction effect of age × gender on the gray matter ratio. A decrease in the volume of gray matter is thought to result from both degenerative and maturational changes. Degenerative changes in gray matter, including shrinkage or loss of neurons [48] and the loss of dendritic arborization [49], have been considered to be involved in the decline of gray matter volume. On the other hand, brain maturation, consisting of both regressive cellular events, such as synaptic pruning, and progressive cellular events, such as myelination, occurs simultaneously in the brain during childhood, adolescence, and young adulthood. Both types of events might result in the decline of regional gray matter volume or cortical thinning on MR images [50]. Thus, the decrease in gray matter ratio observed on MR images is thought to reflect both degenerative and maturational changes in the gray matter.

Gender differences are known to exist in the way gray matter changes during the course of brain maturation. Indeed, the volumes of the frontal and parietal gray matter peak earlier in females [51], and the slope of the reduction in gray matter volume in adolescence is steeper in males than in females [52]. Additionally, estradiol delayed synaptic pruning in an animal study [53], suggesting a process that could slow the decrease in gray matter volume, in females. In contrast, testosterone has been thought to be associated with myelinogenesis [54], a mechanism that could lead to the enhanced decline in gray matter volume in men. For these reasons, results indicating that men have a significantly higher gray matter ratio, and indicating that young adult men have a steeper decrease in the volume of gray matter than young adult women, might be explained by differences in both sex hormones and delayed maturation in males relative to females.

Regarding degenerative changes in gray matter, previous studies relevant to gender differences in brain degeneration have shown that hormone replacement therapy in postmenopausal women was associated with a sparing of gray matter volume, suggesting that estrogen might have neuroprotective effects [36], [37]. Additionally, the average age of menopause has been estimated at about 50 years in Japanese women [55], suggesting that the slope of the regression line between age and gray matter volume in women younger than about 50 years of age might not be steeper than that in women after menopause. Additionally, recent studies and our results have shown that white matter volume increased until the fifth decade of life and subsequently declined [9], [56]. Considering the inverted U-shaped curvilinear trajectory of the white matter volume as a function of age, the decrease in the volume of gray matter until about the fifth decade of life is thought to reflect primarily maturational changes, whereas that from about the sixth decade on is thought to reflect primarily degenerative changes. Additionally, because several cerebrovascular risk factors, such as hypertension or elevation of systolic blood pressure [12], [57]–[59], excessive alcohol drinking [38], [60], and obesity [39], [61]–[63], are associated with a decrease in gray matter volume, these factors may affect degeneration of the gray matter.

On the basis of these results, in women, the reason that the correlation between age and gray matter ratio was significant and was best fit by a second-order polynomial function is thought to be due to the combination of the neuroprotective effect of estrogen tending to decrease the loss of gray matter, particularly in younger women, and the degenerative effect of aging in older women. On the other hand, the reason that the significant correlation between age and gray matter volume in men was best fit by a first-order polynomial function appears to be due to the combined effect of the delayed maturation of men versus women, and the tendency of this maturation to decrease gray matter volume in younger men, with the degenerative effect of aging leading to the continued decline in gray matter in older men.

### 2. Regional gray matter volume

#### 2.1. Main effect of age

All gray matter regions showed significant main effects of age, with age-related volume decline in all gray matter regions. Several regions, in particular, the precentral gyrus, postcentral gyrus, middle frontal gyrus, and insula, showed significant and larger main effects for age on gray matter volume and steeper loss of this volume with aging than the other brain regions. These results are consistent with those of recent studies demonstrating that several gray matter regions, including the perisylvian regions and prefrontal lobe, were significantly negatively correlated with age [1], [5], [6], [12]. On the other hand, consistent with previous studies [6], [9], other regions, such as the parahippocampal gyrus, thalamus, cingulate cortex, and occipital lobe, showed relatively minimal declines with aging in gray matter volumes. Although the mechanism underlying regional differences in age-related gray matter volume decline has not been identified, myelination and the presence of cerebrovascular risk factors might partially account for this phenomenon. Regarding myelination, in the association cortex of the frontal lobe myelination continues into adulthood [64], suggesting that the gray matter volume loss in these regions observed in the MR images of young adults might be significant. On the other hand, the slight degree of myelination that appears in the anterior and posterior cingulate cortices during the second decade and remains relatively constant throughout subsequent decades [64], and the myelination completed in early childhood in the occipital lobe [64], suggest that the gray matter volume loss in these regions observed in MR images of young adults may be minimal. However, because we did not collect images related to the extent of myelination, such as magnetization transfer ratio images [65]–[68], we cannot show the relationship between myelination and gray matter volume. Regarding cerebrovascular risk factors, a decline in frontal lobe gray matter volume is associated with excessive alcohol drinking [38], [60] and hypertension [59]. Thus, the greater loss of gray matter volume in the frontal lobe than in other regions may be associated with these cerebrovascular risk factors.

#### 2.2. Interaction of age × gender

In the ROI analysis, most regions, with the exception of the inferior parietal lobule, orbital gyrus, supramarginal gyrus, and posterior lobe of the cerebellum, showed significant age × gender interactions. These results indicated that the trajectory along which the gray matter volume decreased as a function of aging differed between men and women in almost all regions. In particular, the gray matter regions of the basal aspect of the temporal and occipital lobes, including the fusiform gyrus, inferior occipital gyrus, parahippocampal gyrus, and lingual gyrus, showed greater effects of the age × gender interaction than did other regions. Additionally, we showed that the bilateral lateral frontal gyrus showed a significant age × gender interaction, whereas the bilateral temporoparietal cortex showed no significant age × gender interaction. These results are consistent with the findings of a recent study that applied cortical thickness analysis [31]. In addition to these findings, we showed that there is a significant age × gender interaction in the volume of gray matter in the caudate. This finding is consistent with a recent study, which showed that the age-related decrease in caudate volume was greater in females than in males [69]. Thus, most gray matter regions showed significant age × gender interactions. However, recent studies have shown no significant gender differences in the correlation between regional gray matter volume and age [13], [16]. The inconsistency of our results and those of recent studies derives from differences in the age ranges of the subjects or in the regions under study. For example, one recent study analyzed elderly subjects aged 63–75 years [16]. Because sex hormones, such as estrogen, are thought to be involved in gender differences in the decrease in gray matter with age, the impact of these hormones might have been lower among subjects in this age group than among the subjects in our study. Additionally, the regions analyzed in the other recent study consisted primarily of such large areas as the frontal lobes [13], whereas we analyzed smaller regions corresponding to gyral structures. Furthermore, the number of the subjects in our study was much larger than the number included in the other studies. For these reasons, we believe that our findings can be generalized to healthy individuals from young adulthood to early older age.

The mechanisms underlying age × gender interactions are thought to be associated with the gender differences accompanying maturational changes, degenerative changes, and the effect of several cerebrovascular risk factors, as described in section 4.1. Regarding maturational changes, a previous study has shown that gray matter reached maximal volume about 1 year earlier in women than in men in the frontal and parietal lobes, suggesting that these brain regions mature earlier in women than in men [51]. Additionally, the slope of the reduction in gray matter volume in adolescence is steeper in males than in females [52] due to delayed brain maturation in males relative to females. In our study of several brain regions, we found a steeper decline in gray matter volume in males than in females from the third to the fourth decade of age (Fig. 9, 10).

Regarding degenerative changes in gray matter, the effect of sex hormones, including the neuroprotective effects of estrogen, might substantially affect the age × gender interaction [36], [37]. Additionally, the effects of cerebrovascular risk factors and alcohol consumption have been associated with gray matter volume reduction in specific regions, such as the frontal lobe [38], [60], [70], and these reductions in gray matter volume have been observed only in men [12], [38]. Additionally, obesity has been associated with decreased gray matter volume in men but not in women [39]. Indeed, the prevalence of a past or present drinking habit was significantly higher in male than in female subjects, and the body mass index of male subjects was significantly higher than that of female subjects (Table 1). Thus, it is thought that the smaller decline in gray matter volume in females than in males is due to the neuroprotective effects of estrogen and of the presence of fewer cerebrovascular risk factors in women. Indeed, we found less loss in the volume of gray matter in most brain regions in females than in males, especially during the third through sixth decades of age (Fig. 9, 10).

#### 2.3. The interaction of age × hemisphere

Although several regions such as the superior and middle occipital gyri, showed significant leftward asymmetry (left > right), and several regions such as the cuneus and superior frontal gyrus showed significant rightward asymmetry (right > left), partially consistent with the results of recent studies [71], we found no region that showed a significant age × hemisphere interaction in the ROI analysis. However, we found that several regions, such as the insula, prefrontal cortex, and the posterior lobe of the cerebellum, showed significant age × hemisphere interaction in the voxel-based analysis. The reason for the inconsistency between the results of the ROI and voxel-based analyses may be the distribution of the significant regions. In other words, the regions that had a significant age × hemisphere interaction did not correspond to gyral anatomy. For this reason, we think that we did not detect a significant age × hemisphere interaction in the ROI analysis. Our results suggest that the corresponding gray matter regions in the left and right hemispheres show similar trajectories in their volume decline as a function of aging, and that the hemispheric difference is maintained throughout life, except several regions such as the insula, prefrontal cortex, and the posterior lobe of the cerebellum.

#### 2.4. Interaction of age × gender × hemisphere

The inferior temporal gyrus showed significant age × gender × hemisphere interactions in the ROI analysis. In the voxel-based analysis, only a few small regions, such as the hippocampus and the inferomedial occipital lobe showed significant age × gender × hemisphere interaction. Therefore, we focused on the results of the ROI analysis, in which the inferior temporal gyrus showed a significant age × gender × hemisphere interaction. More specifically, the left inferior temporal gyrus showed significant age × gender interaction, whereas the right inferior temporal gyrus showed no significant age × gender interaction. A recent study has reported regional gender dimorphism in the inferior temporal gyrus [72]. Additionally, men have a larger inferior temporal gyrus than women, and the inferior temporal cortex of men has been reported to be more vulnerable to shrinkage with aging than that of women, suggesting that the age × gender interaction has an effect in this region [32]. Additionally, the inferior temporal cortex is one of the regions showing significant hemispheric differences in volume [32]. We confirmed these findings by observing an age × gender × hemisphere interaction in the inferior temporal gyrus. However, one study reported no significant age × gender × hemisphere interaction in the temporal lobe [73]. This inconsistency is thought to result from the manner in which the temporal lobe was divided. The temporal lobe was divided into anterior, middle, and posterior portions in the previous study, whereas this study divided the temporal lobe according to such anatomical structures as the superior, middle, and inferior temporal gyri. Although we have not clarified the mechanisms underlying the age × gender × hemisphere interaction in the inferior temporal gyrus, our results can be correlated with gender and hemispheric differences in the functional localization of the inferior temporal gyrus. For example, a main effect of gender was found in the left inferior temporal gyrus on a verbal fluency task [74]. Further studies are needed to reveal the mechanisms underlying the effects of age × gender × hemisphere interactions on the regional gray matter volume of the inferior temporal gyrus.

This study has several limitations. First, as described in the Introduction, the present study is a cross-sectional study; that is, we have shown a relationship between regional gray matter volume and age, but we have not shown their relationship over time. However, it would be difficult to analyze the correlation between gray matter volume and age using a longitudinal design with such a large number of subjects (>1000) covering a wide age range. Second, we recruited subjects by announcing the purpose of our study in the mass media; thus, there may be some selection bias, such as by health status. Third, there is a possibility of misclassification during tissue segmentation, such as the classification of white matter hyperintensities as gray matter. Because white matter hyperintensities are common in later life [75], such misclassifications would lead to overestimation of gray matter volume and would tend to diminish age-related declines in gray matter volume. Although we cannot rule out such misclassification in tissue segmentation using our fully automated method, we tried to reduce the possibility by using not only voxel intensity, but also *a priori* knowledge of the normal location of gray matter, white matter, and CSF to instruct the segmentation process. Fourth, as discussed earlier, there is a possibility that potential confounding factors may affect the correlation between gray matter volume and age, because several factors such as elevated systolic blood pressure [12] and excessive alcohol consumption [38], [60] affect regional gray matter volume. Thus, we cannot deny the possibility that the effect of aging on gray matter volume is overestimated due to the presence of those factors. Considering these limitations, we believe the fully automated method of MR image processing was actually a strength in dealing with the large quantity of data objectively and efficiently. Fifth, the voxel-size of the T1-weighted images was 1.02×1.02×1.5 mm; therefore, it is possible that the partial-volume effect affected the process of tissue segmentation. To decrease that possibility, we checked all gray matter segments to evaluate whether there was a misclassification by visual inspection. Although we cannot deny the possibility, no obvious misclassification was found in the segmented gray matter images.

In conclusion, we examined correlations between global and regional gray matter volumes and age and the interaction of gender and hemisphere using magnetic resonance images obtained from 1460 healthy individuals over a wide age range. In analyzing global gray matter volume, a significant negative correlation was found between gray matter ratio and age in both genders, and a significant interaction effect of age × gender on gray matter ratio also emerged. In analyzing regional gray matter volume, the gray matter volume of all regions showed significant main effects of age, and almost all regions, except the inferior parietal lobule, showed a significant age × gender interaction. Additionally, the inferior temporal gyrus showed a significant age × gender × hemisphere interaction. No region showed a significant age × hemisphere interactions. Our study may contribute not only to clarifying the mechanism(s) of normal brain aging in each brain region, but also to distinguishing normal from pathological aging.

## Supporting Information

Table S1 **Mean and standard deviation of regional gray matter volume in each parcel, and percentage annual decrease in regional gray matter volume estimated by the linear regression correlation between regional gray matter volume and age.**
(DOC)

Table S2 **Correlations between regional gray matter volume in each structure and age estimated using first-, second-, and third-order polynomial functions.**
(DOC)

Table S3 **Gray matter regions and coordinates of Talairach space of local maxima, showing significant main effect of age.**
(DOC)

Table S4 **Gray matter regions and coordinates of Talairach space of local maxima, showing significant age × gender interaction.**
(DOC)

Table S5 **Gray matter regions and coordinates of Talairach space of local maxima, showing significant age × hemisphere interaction.**
(DOC)

Table S6 **Gray matter regions and coordinates of Talairach space of local maxima, showing significant age × gender × hemisphere interaction.**
(DOC)

Table S7 **Effects of age, gender, and hemisphere, and interactions of age × gender, age × hemisphere, and age × gender × hemisphere on regional gray matter volume in each structure by effect size using partial** ***η*** **^2^.** Intracranial volume is used as a covariate. In the gender column, bold type represents significantly larger gray matter volume in the region among women than among men. In the hemisphere column, bold type represents significant leftward asymmetry (left > right) in the gray matter volume in the region.
(DOC)
