# Supplementary material for: Correlations among Brain Gray Matter Volumes, Age, Gender, and Hemisphere in Healthy Individuals
Source: PLoS One. 2011 Jul 27;6(7):e22734. doi: 10.1371/journal.pone.0022734 (PMC3144937; doi:10.1371/journal.pone.0022734)
Supplement: Table S1 — Mean and standard deviation of regional gray matter volume in each parcel, and percentage annual decrease in regional gray matter volume estimated by the linear regression correlation between regional gray matter volume and age. (DOC) [file pone.0022734.s001.doc]

Table S1. Mean and standard deviation of regional gray matter volume in each parcel, and percentage annual decrease in regional gray matter volume estimated by the linear regression correlation between regional gray matter volume and age.

| Structure | Men |  |  |  | Women |  |  |  |
| --- | --- | --- | --- | --- | --- | --- | --- | --- |
|  | Left |  | Right |  | Left |  | Right |  |
|  | Mean (*SD*) | Decrease  (%/year) | Mean (*SD*) | Decrease  (%/year) | Mean (*SD*) | Decrease  (%/year) | Mean (*SD*) | Decrease  (%/year) |
| Angular gyrus | 0.322 (0.051) | 0.141 | 0.311 (0.049) | 0.131 | 0.330 (0.045) | 0.174 | 0.315 (0.042) | 0.155 |
| Anterior cingulate cortex | 0.334 (0.037) | 0.110 | 0.341 (0.038) | 0.104 | 0.294 (0.031) | 0.089 | 0.300 (0.031) | 0.084 |
| Caudate nucleus | 0.273 (0.035) | 0.115 | 0.281 (0.035) | 0.098 | 0.264 (0.029) | 0.097 | 0.271 (0.029) | 0.087 |
| Cingulate cortex | 0.321 (0.036) | 0.079 | 0.324 (0.036) | 0.071 | 0.285 (0.031) | 0.062 | 0.289 (0.032) | 0.057 |
| Cuneus | 0.319 (0.035) | 0.061 | 0.304 (0.034) | 0.045 | 0.290 (0.031) | 0.099 | 0.275 (0.029) | 0.091 |
| Fusiform gyrus | 0.468 (0.062) | 0.182 | 0.452 (0.063) | 0.189 | 0.404 (0.039) | 0.073 | 0.389 (0.039) | 0.074 |
| Inferior frontal gyrus | 0.333 (0.035) | 0.110 | 0.332 (0.036) | 0.126 | 0.300 (0.029) | 0.103 | 0.297 (0.029) | 0.113 |
| Inferior occipital gyrus | 0.273 (0.053) | 0.158 | 0.258 (0.049) | 0.143 | 0.233 (0.026) | 0.038 | 0.218 (0.024) | 0.041 |
| Inferior parietal lobule | 0.311 (0.045) | 0.183 | 0.316 (0.044) | 0.176 | 0.299 (0.037) | 0.158 | 0.305 (0.037) | 0.151 |
| Inferior temporal gyrus | 0.464 (0.054) | 0.141 | 0.508 (0.058) | 0.092 | 0.409 (0.040) | 0.060 | 0.387 (0.038) | 0.070 |
| Insula | 0.379 (0.048) | 0.176 | 0.378 (0.051) | 0.197 | 0.354 (0.042) | 0.179 | 0.353 (0.044) | 0.194 |
| Lingual gyrus | 0.383 (0.058) | 0.169 | 0.375 (0.056) | 0.143 | 0.315 (0.032) | 0.059 | 0.307 (0.032) | 0.049 |
| Medial superior frontal gyrus | 0.425 (0.046) | 0.114 | 0.425 (0.046) | 0.107 | 0.374 (0.036) | 0.110 | 0.372 (0.036) | 0.104 |
| Middle frontal gyrus | 0.284 (0.036) | 0.157 | 0.287 (0.037) | 0.165 | 0.251 (0.028) | 0.111 | 0.253 (0.028) | 0.115 |
| Middle occipital gyrus | 0.283 (0.033) | 0.078 | 0.260 (0.030) | 0.071 | 0.259 (0.025) | 0.057 | 0.238 (0.022) | 0.053 |
| Middle temporal gyrus | 0.418 (0.040) | 0.075 | 0.415 (0.039) | 0.086 | 0.383 (0.035) | 0.070 | 0.375 (0.033) | 0.072 |
| Orbital gyrus | 0.306 (0.040) | 0.097 | 0.322 (0.042) | 0.102 | 0.261 (0.028) | 0.082 | 0.273 (0.030) | 0.088 |
| Paracentral lobule | 0.334 (0.047) | 0.123 | 0.325 (0.046) | 0.126 | 0.292 (0.033) | 0.078 | 0.286 (0.033) | 0.082 |
| Parahippocampal gyrus | 0.474 (0.049) | 0.120 | 0.472 (0.051) | 0.136 | 0.397 (0.034) | 0.045 | 0.392 (0.034) | 0.042 |
| Posterior cingulate cortex | 0.393 (0.051) | 0.035 | 0.408 (0.056) | 0.028 | 0.343 (0.041) | 0.062 | 0.358 (0.043) | 0.064 |
| Postcentral gyrus | 0.274 (0.038) | 0.160 | 0.278 (0.038) | 0.161 | 0.251 (0.029) | 0.118 | 0.256 (0.030) | 0.119 |
| Precentral gyrus | 0.264 (0.037) | 0.167 | 0.266 (0.037) | 0.169 | 0.239 (0.027) | 0.123 | 0.242 (0.029) | 0.126 |
| Precuneus | 0.346 (0.042) | 0.115 | 0.341 (0.042) | 0.112 | 0.319 (0.035) | 0.123 | 0.312 (0.035) | 0.124 |
| Rectal gyrus | 0.425 (0.054) | 0.111 | 0.434 (0.055) | 0.113 | 0.356 (0.036) | 0.096 | 0.362 (0.037) | 0.095 |
| Superior frontal gyrus | 0.283 (0.034) | 0.128 | 0.294 (0.036) | 0.133 | 0.251 (0.026) | 0.096 | 0.261 (0.027) | 0.101 |
| Superior occipital gyrus | 0.308 (0.047) | 0.099 | 0.270 (0.042) | 0.085 | 0.311 (0.042) | 0.147 | 0.268 (0.037) | 0.123 |
| Superior temporal gyrus | 0.387 (0.043) | 0.118 | 0.395 (0.042) | 0.106 | 0.359 (0.038) | 0.123 | 0.364 (0.036) | 0.120 |
| Superior parietal lobule | 0.301 (0.047) | 0.172 | 0.297 (0.047) | 0.168 | 0.274 (0.034) | 0.134 | 0.268 (0.034) | 0.129 |
| Supramerginal gyrus | 0.316 (0.047) | 0.140 | 0.324 (0.045) | 0.128 | 0.319 (0.040) | 0.146 | 0.328 (0.041) | 0.144 |
| Thalamus | 0.307 (0.037) | 0.067 | 0.319 (0.039) | 0.069 | 0.298 (0.032) | 0.038 | 0.310 (0.033) | 0.038 |
| Anterior lobe of the cerebellum | 0.438 (0.061) | 0.181 | 0.442 (0.061) | 0.170 | 0.386 (0.046) | 0.081 | 0.391 (0.048) | 0.070 |
| Posterior lobe of the cerebellum | 0.452 (0.049) | 0.108 | 0.453 (0.050) | 0.080 | 0.390 (0.041) | 0.115 | 0.388 (0.040) | 0.105 |
