# Supplementary material for: Correlations among Brain Gray Matter Volumes, Age, Gender, and Hemisphere in Healthy Individuals
Source: PLoS One. 2011 Jul 27;6(7):e22734. doi: 10.1371/journal.pone.0022734 (PMC3144937; doi:10.1371/journal.pone.0022734)
Supplement: Table S2 — Correlations between regional gray matter volume in each structure and age estimated using first-, second-, and third-order polynomial functions. (DOC) [file pone.0022734.s002.doc]

Table S2. Correlations between regional gray matter volume in each structure and age estimated using first-, second-, and third-order polynomial functions.

| Structure | Men |  |  |  | Women |  |  |  |
| --- | --- | --- | --- | --- | --- | --- | --- | --- |
|  | Left |  | Right |  | Left |  | Right |  |
|  | Function | Adjusted *R^2^* | Function | Adjusted *R^2^* | Function | Adjusted *R^2^* | Function | Adjusted *R^2^* |
| Angular gyrus | Linear | .170 | Linear | .158 | Linear | .261 | Linear | .235 |
| Anterior cingulate cortex | Cubic | .203 | Cubic | .181 | Linear | .149 | Quadratic | .131 |
| Caudate nucleus | Linear | .235 | Linear | .168 | Linear | .196 | Cubic | .175 |
| Cingulate cortex | Quadratic | .144 | Quadratic | .124 | Linear | .067 | Linear | .055 |
| Cuneus | Quadratic | .070 | Quadratic | .045 | Linear | .183 | Linear | .169 |
| Fusiform gyrus | Quadratic | .209 | Quadratic | .225 | Quadratic | .086 | Quadratic | .091 |
| Inferior frontal gyrus | Linear | .223 | Linear | .276 | Cubic | .243 | Cubic | .278 |
| Inferior occipital gyrus | Quadratic | .222 | Quadratic | .214 | Quadratic | .056 | Quadratic | .076 |
| Inferior parietal lobule | Quadratic | .364 | Quadratic | .351 | Linear | .326 | Linear | .296 |
| Inferior temporal gyrus | Linear | .148 | Linear | .054 | Cubic | .061 | Cubic | .075 |
| Insula | Linear | .294 | Linear | .335 | Linear | .318 | Linear | .350 |
| Lingual gyrus | Quadratic | .202 | Quadratic | .160 | Quadratic | .081 | Quadratic | .071 |
| Medial superior frontal gyrus | Cubic | .143 | Cubic | .126 | Quadratic | .169 | Quadratic | .152 |
| Middle frontal gyrus | Cubic | .444 | Cubic | .457 | Cubic | .302 | Cubic | .310 |
| Middle occipital gyrus | Quadratic | .125 | Linear | .123 | Cubic | .115 | Cubic | .114 |
| Middle temporal gyrus | Quadratic | .079 | Linear | .108 | Linear | .071 | Linear | .081 |
| Orbital gyrus | Cubic | .135 | Quadratic | .138 | Linear | .151 | Linear | .153 |
| Paracentral lobule | Quadratic | .185 | Quadratic | .203 | Linear | .096 | Linear | .110 |
| Parahippocampal gyrus | Cubic | .140 | Quadratic | .171 | Quadratic | .038 | Quadratic | .051 |
| Posterior cingulate cortex | Linear | .009 | Linear | .004 | Linear | .039 | Cubic | .047 |
| Postcentral gyrus | Cubic | .411 | Cubic | .402 | Cubic | .309 | Cubic | .290 |
| Precentral gyrus | Cubic | .470 | Cubic | .467 | Cubic | .363 | Cubic | .349 |
| Precuneus | Quadratic | .179 | Quadratic | .174 | Quadratic | .224 | Quadratic | .231 |
| Rectal gyrus | Cubic | .100 | Cubic | .103 | Quadratic | .130 | Quadratic | .124 |
| Superior frontal gyrus | Cubic | .337 | Quadratic | .332 | Cubic | .263 | Quadratic | .272 |
| Superior occipital gyrus | Linear | .097 | Linear | .090 | Linear | .217 | Linear | .196 |
| Superior temporal gyrus | Quadratic | .167 | Quadratic | .140 | Linear | .187 | Linear | .188 |
| Superior parietal lobule | Quadratic | .306 | Quadratic | .292 | Quadratic | .274 | Cubic | .264 |
| Supramerginal gyrus | Linear | .199 | Linear | .179 | Linear | .230 | Linear | .219 |
| Thalamus | Linear | .071 | Quadratic | .069 | Linear | .024 | Linear | .021 |
| Anterior lobe of the cerebellum | Quadratic | .216 | Quadratic | .193 | Quadratic | .071 | Quadratic | .053 |
| Posterior lobe of the cerebellum | Quadratic | .111 | Quadratic | .063 | Quadratic | .146 | Quadratic | .124 |

All *R^2^* were significant when Bonferroni-corrected for multiple comparisons, *p* < 0.002 except the bilateral posterior cingulate cortices in men (left, *p* = .006; right, *p* = .045)
