# Supplementary material for: Correlations among Brain Gray Matter Volumes, Age, Gender, and Hemisphere in Healthy Individuals
Source: PLoS One. 2011 Jul 27;6(7):e22734. doi: 10.1371/journal.pone.0022734 (PMC3144937; doi:10.1371/journal.pone.0022734)
Supplement: Table S3 — Gray matter regions and coordinates of Talairach space of local maxima, showing significant main effect of age. (DOC) [file pone.0022734.s003.doc]

Table S3. Gray matter regions and coordinates of Talairach space of local maxima, showing significant main effect of age.

| Location^*^ | *x* | *y* | *z* | *F* | *p* |
| --- | --- | --- | --- | --- | --- |
| R insula | 45 | −7 | −0 | 1182.99 | < 0.001 |
| R thalamus | 16 | −16 | 12 | 185.13 | < 0.001 |
| R uncus | 24 | −2 | −27 | 185.06 | < 0.001 |
| L uncus | −22 | −3 | −26 | 177.97 | < 0.001 |
| L thalamus | −15 | −16 | 12 | 143.91 | < 0.001 |
| R cerebellum (posterior lobe) | 20 | −60 | −50 | 51.87 | < 0.001 |
| L superior temporal gyrus | −54 | 12 | −17 | 50.27 | < 0.001 |
| R cerebellum (anterior lobe) | 1 | −49 | −11 | 44.99 | < 0.001 |
| L cerebellum (posterior lobe) | −22 | −56 | −51 | 42.08 | < 0.001 |
| L middle temporal gyrus | −69 | −22 | −6 | 38.67 | < 0.001 |

^*^: To summarize the results, the regions whose cluster size is more than 100 were shown.
