# Supplementary material for: Correlations among Brain Gray Matter Volumes, Age, Gender, and Hemisphere in Healthy Individuals
Source: PLoS One. 2011 Jul 27;6(7):e22734. doi: 10.1371/journal.pone.0022734 (PMC3144937; doi:10.1371/journal.pone.0022734)
Supplement: Table S4 — Gray matter regions and coordinates of Talairach space of local maxima, showing significant age × gender interaction. (DOC) [file pone.0022734.s004.doc]

Table S4. Gray matter regions and coordinates of Talairach space of local maxima, showing significant age × gender interaction.

| Location | *x* | *y* | *z* | *F* | *p* |
| --- | --- | --- | --- | --- | --- |
| R cerebellum (anterior lobe) | 9 | −28 | −15 | 143.16 | < 0.001 |
| R cerebellum (posterior lobe) | 16 | −53 | −65 | 88.52 | < 0.001 |
| L precentral gyrus | −37 | −12 | 53 | 78.71 | < 0.001 |
| L cerebellum (posterior lobe) | −28 | −49 | −47 | 75.25 | < 0.001 |
| R precentral gyrus | 46 | −9 | −52 | 63.32 | < 0.001 |
| L cuneus | −1 | −87 | 24 | 50.04 | < 0.001 |
| L inferior temporal gyrus | −49 | −25 | −15 | 49.58 | < 0.001 |
| L cingulate gyrus | −18 | 17 | 25 | 46.22 | < 0.001 |
| R cuneus | 28 | −84 | 23 | 41.54 | < 0.001 |
| R uncus | 34 | 9 | −48 | 41.51 | < 0.001 |
| R superior frontal gyrus | 4 | 35 | 47 | 35.92 | < 0.001 |
| L parahippocampal gyrus | −35 | −43 | 1 | 34.23 | 0.001 |
| R caudate nucleus | 25 | −2 | 28 | 34.21 | 0.001 |
| R uncus | 27 | 25 | −33 | 32.33 | 0.001 |
| R superior parietal lobule | 19 | −48 | 58 | 32.17 | 0.001 |
| R superior frontal gyrus | 27 | 10 | 69 | 30.52 | 0.003 |
| L middle occipital gyrus | −33 | −98 | 7 | 30.36 | 0.003 |
| L superior frontal gyrus | 0 | 49 | 23 | 29.05 | 0.006 |
| L cuneus | −23 | −86 | 27 | 28.62 | 0.007 |
| R superior temporal gyrus | 59 | −49 | 17 | 27.66 | 0.011 |
| R superior temporal gyrus | 51 | −28 | 2 | 26.34 | 0.019 |

^*^: To summarize the results, the regions whose cluster size is more than 100 were shown.
