# Supplementary material for: Correlations among Brain Gray Matter Volumes, Age, Gender, and Hemisphere in Healthy Individuals
Source: PLoS One. 2011 Jul 27;6(7):e22734. doi: 10.1371/journal.pone.0022734 (PMC3144937; doi:10.1371/journal.pone.0022734)
Supplement: Table S5 — Gray matter regions and coordinates of Talairach space of local maxima, showing significant age × hemisphere interaction. (DOC) [file pone.0022734.s005.doc]

Table S5. Gray matter regions and coordinates of Talairach space of local maxima, showing significant age × hemisphere interaction.

| Location | *x* | *y* | *z* | *F* | *p* |
| --- | --- | --- | --- | --- | --- |
| Thalamus | 3 | −14 | 5 | 420.64 | < 0.001 |
| Insula | 46 | −4 | 10 | 206.80 | < 0.001 |
| Cerebellum (anterior lobe) | 6 | −31 | −6 | 139.58 | < 0.001 |
| Cerebellum (posterior lobe) | 18 | −39 | −48 | 125.14 | < 0.001 |
| Cingulate gyrus | 4 | 6 | 34 | 102.06 | < 0.001 |
| Precentral gyrus | 45 | −18 | 32 | 85.71 | < 0.001 |
| Cerebellum (posterior lobe) | 12 | −68 | −52 | 82.06 | < 0.001 |
| Precuneus | 1 | −47 | 63 | 79.21 | < 0.001 |
| Lingual gyrus | 1 | −97 | −10 | 71.74 | < 0.001 |
| Cuneus | 1 | −80 | 26 | 63.24 | < 0.001 |
| Cerebellum (posterior lobe) | 8 | −45 | −38 | 60.90 | < 0.001 |
| Anterior cingulate cortex | 17 | 39 | 6 | 54.43 | < 0.001 |
| Superior frontal gyrus | 12 | 65 | −23 | 54.05 | < 0.001 |
| Cingulate gyrus | 25 | −17 | 46 | 53.73 | < 0.001 |
| Middle temporal gyrus | 56 | −16 | −9 | 51.29 | < 0.001 |
| Precuneus | 7 | −68 | 27 | 49.57 | < 0.001 |
| Cerebellum (posterior lobe) | 2 | −56 | −11 | 49.00 | < 0.001 |
| Inferior parietal lobule | 55 | −32 | 41 | 47.51 | < 0.001 |
| Uncus | 22 | 6 | −21 | 46.89 | < 0.001 |
| Superior temporal gyrus | 38 | 12 | −18 | 44.63 | < 0.001 |
| Superior temporal gyrus | 52 | 8 | −14 | 44.05 | < 0.001 |
| Middle occipital gyrus | 28 | −90 | 20 | 41.57 | < 0.001 |
| Cerebellum (posterior lobe) | 36 | −41 | −36 | 39.10 | < 0.001 |
| Anterior cingulate cortex | 9 | 28 | 4 | 37.83 | < 0.001 |

^*^: To summarize the results, the regions whose cluster size is more than 100 were shown.
