# Supplementary material for: Correlations among Brain Gray Matter Volumes, Age, Gender, and Hemisphere in Healthy Individuals
Source: PLoS One. 2011 Jul 27;6(7):e22734. doi: 10.1371/journal.pone.0022734 (PMC3144937; doi:10.1371/journal.pone.0022734)
Supplement: Table S6 — Gray matter regions and coordinates of Talairach space of local maxima, showing significant age × gender × hemisphere interaction. (DOC) [file pone.0022734.s006.doc]

Table S6. Gray matter regions and coordinates of Talairach space of local maxima, showing significant age × gender × hemisphere interaction.

| Location | *x* | *y* | *z* | *F* | *p* |
| --- | --- | --- | --- | --- | --- |
| Parahippocampal gyrus | 27 | −28 | −9 | 43.81 | < 0.001 |
| Lingual gyrus | 8 | −95 | −12 | 38.61 | < 0.001 |
| Cerebellum (posterior lobe) | 41 | −73 | −45 | 35.55 | < 0.001 |
| Parahippocampal gyrus | 27 | −50 | −5 | 35.11 | < 0.001 |
| Fusiform gyrus | 43 | −23 | −15 | 34.92 | 0.001 |
| Cerebellum (anterior lobe) | 11 | −26 | −21 | 30.94 | 0.003 |

^*^: To summarize the results, the regions whose cluster size is more than 100 were shown.
