# Supplementary material for: Correlations among Brain Gray Matter Volumes, Age, Gender, and Hemisphere in Healthy Individuals
Source: PLoS One. 2011 Jul 27;6(7):e22734. doi: 10.1371/journal.pone.0022734 (PMC3144937; doi:10.1371/journal.pone.0022734)
Supplement: Table S7 — Effects of age, gender, and hemisphere, and interactions of age × gender, age × hemisphere, and age × gender × hemisphere on regional gray matter volume in each structure by effect size using partial η 2. Intracranial volume is used as a covariate. In the gender column, bold type represents significantly larger gray matter volume in the region among women than among men. In the hemisphere column, bold type represents significant leftward asymmetry (left > right) in the gray matter volume in the region. (DOC) [file pone.0022734.s007.doc]

Table S7. Effects of age, gender, and hemisphere, and interactions of age × gender, age × hemisphere, and age × gender × hemisphere on regional gray matter volume in each structure by effect size using partial *η*^2^. Intracranial volume is used as a covariate. In the gender column, bold type represents significantly larger gray matter volume in the region among women than among men. In the hemisphere column, bold type represents significant leftward asymmetry (left > right) in the gray matter volume in the region.

| Structure | Graph^a^ | Effect size |  |  |  |  |  |
| --- | --- | --- | --- | --- | --- | --- | --- |
|  |  | Age (A) | Gender (G) | Hemisphere (H) | A × G | A × H | A ×G × H |
| Precentral gyrus | Fig. 9, (B) | 0.514^**^ | **0.008**^**^ | 0.003^*^ | 0.049^**^ | 0.003 | 0.003 |
| Middle frontal gyrus | Fig. 9, (G) | 0.504^**^ | 0.001 | 0.004^**^ | 0.053^**^ | 0.002 | 0.003 |
| Postcentral gyrus | Fig. 9, (D) | 0.444^**^ | **0.019**^**^ | 0.010^**^ | 0.042^**^ | 0.004 | 0.003 |
| Insula | Fig. 10, (G) | 0.441^**^ | **0.064**^**^ | 0 | 0.038^**^ | 0.004 | 0.002 |
| Inferior parietal lobule | Fig. 9, (F) | 0.418^**^ | **0.105**^**^ | 0.011^**^ | 0.025^*^ | 0.004 | 0.002 |
| Superior frontal gyrus | Fig. 9, (A) | 0.400^**^ | 0 | 0.076^**^ | 0.039^**^ | 0.002 | 0.002 |
| Inferior frontal gyrus | Fig. 9, (L) | 0.377^**^ | 0 | 0.003^*^ | 0.040^**^ | 0.005 | 0.002 |
| Anterior cingulate cortex | Fig. 10, (F) | 0.321^**^ | **0.009**^**^ | 0.033^**^ | 0.040^**^ | 0.001 | 0.001 |
| Superior parietal lobule | Fig. 9, (C) | 0.313^**^ | 0.001 | **0.005**^**^ | 0.033^**^ | 0.002 | 0.003 |
| Superior temporal gyrus | Fig. 9, (J) | 0.286^**^ | **0.049**^**^ | 0.018^**^ | 0.039^**^ | 0.002 | 0.003 |
| Supramerginal gyrus | Fig. 9, (E) | 0.283^**^ | **0.213**^**^ | 0.016^**^ | 0.025^*^ | 0.005 | 0.003 |
| Precuneus | Fig. 10, (C) | 0.277^**^ | **0.045**^**^ | **0.012**^**^ | 0.042^**^ | 0.002 | 0.001 |
| Angular gyrus | Fig. 9, (I) | 0.258^**^ | **0.188**^**^ | **0.031**^**^ | 0.032^**^ | 0.004 | 0.004 |
| Medial superior frontal gyrus | Fig. 10, (A) | 0.227^**^ | 0.003^*^ | 0 | 0.031^**^ | 0.001 | 0.001 |
| Caudate nucleus | Fig. 10, (H) | 0.223^**^ | **0.028**^**^ | 0.020^**^ | 0.039^**^ | 0.004 | 0.002 |
| Orbital gyrus | Fig. 10, (L) | 0.181^**^ | 0.035^**^ | 0.060^**^ | 0.029^*^ | 0.002 | 0.003 |
| Paracentral lobule | Fig. 9, (H) | 0.180^**^ | 0.001 | **0.016**^**^ | 0.038^**^ | 0.002 | 0.002 |
| Superior occipital gyrus | Fig. 10, (K) | 0.180^**^ | **0.148**^**^ | **0.257**^**^ | 0.051^**^ | 0.006 | 0.007 |
| Fusiform gyrus | Fig. 10, (O) | 0.174^**^ | 0.033^**^ | **0.035**^**^ | 0.070^**^ | 0.002 | 0.002 |
| Middle temporal gyrus | Fig. 9, (M) | 0.172^**^ | **0.012**^**^ | **0.011**^**^ | 0.044^**^ | 0.003 | 0.006 |
| Middle occipital gyrus | Fig. 9, (K) | 0.160^**^ | **0.009**^**^ | **0.213**^**^ | 0.035^**^ | 0.005 | 0.004 |
| Cuneus | Fig. 10, (E) | 0.157^**^ | **0.011**^**^ | **0.092**^**^ | 0.065^**^ | 0.002 | 0.003 |
| Cingulate cortex | Fig. 10, (D) | 0.152^**^ | **0.024**^**^ | 0.007^**^ | 0.040^**^ | 0.001 | 0.001 |
| Inferior occipital gyrus | Fig. 9, (N) | 0.147^**^ | 0.029^**^ | **0.042**^**^ | 0.069^**^ | 0.003 | 0.004 |
| Lingual gyrus | Fig. 10, (J) | 0.145^**^ | 0.106^**^ | **0.010**^**^ | 0.059^**^ | 0.003 | 0.002 |
| Anterior lobe of the cerebellum | Fig. 9, (Q) | 0.145^**^ | 0.006^**^ | 0.002^*^ | 0.049^**^ | 0.002 | 0.002 |
| Rectal gyrus | Fig. 10, (M) | 0.139^**^ | 0.067^**^ | 0.010^**^ | 0.030^**^ | 0.001 | 0.002 |
| Parahippocampal gyrus | Fig. 10, (N) | 0.131^**^ | 0.147^**^ | 0.002 | 0.060^**^ | 0.004 | 0.004 |
| Posterior lobe of the cerebellum | Fig. 9, (R) | 0.131^**^ | 0.038^**^ | 0 | 0.030^*^ | 0.003 | 0.003 |
| Inferior temporal gyrus | Fig. 9, (O, P) | 0.110^**^ | 0.151^**^ | 0.020^**^ | 0.040^**^ | 0.013 | 0.176^**^ |
| Thalamus | Fig. 10, (I) | 0.070^**^ | **0.034**^**^ | 0.036^**^ | 0.036^**^ | 0.001 | 0.002 |
| Posterior cingulate cortex | Fig. 10, (B) | 0.046^**^ | 0.002^*^ | 0.039^**^ | 0.040^**^ | 0.002 | 0.002 |

^a^: Correspondence with the graphs in Figures 9 and 10

^*^*P* < 0.05; ^**^*P* < 0.002. Bonferroni critical α = 0.002.
